# Dysregulation of the miR-146a-Smad4 axis impairs osteogenesis of bone mesenchymal stem cells under inflammation

**DOI:** 10.1038/boneres.2017.37

**Published:** 2017-11-21

**Authors:** Wei Kuang, Liwei Zheng, Xin Xu, Yao Lin, Jiong Lin, Jiahua Wu, Jiali Tan

**Affiliations:** 1 Department of Stomatology, Guangzhou General Hospital of Guangzhou Military Command, Guangzhou, China; 2 State Key Laboratory of Oral Diseases, Sichuan University, Chengdu, China; 3 State Key Laboratory of Oral Diseases, West China Hospital of Stomatology, Sichuan University, Chengdu, China; 4 Department of Orthodontics, Guanghua School of Stomatology, Hospital of Stomatology, Sun Yat-sen University and Guangdong Provincial Key Laboratory of Stomatology, Guangzhou, China

## Abstract

Osteoporosis is a common disease that affects patient quality of life, especially among the elderly population. Although inflammation contributes significantly to osteoporosis, the underlying mechanism is unclear. In this study, we found that tumor necrosis factor (TNF)-α, an inflammatory environment mimic, inhibits osteogenesis of bone mesenchymal stem cells (BMSCs), induces miR-146a and decreases Smad4. Moreover, overexpression of miR-146a inhibited the osteogenic ability of BMSCs, whereas blocking miR-146a partially rescued the osteogenesis deficiency under TNF*-*α treatment. Molecularly, miR-146a decreased Smad4 expression at the protein level by binding to an element located in the Smad4 3′-untranslated region, and restoration of Smad4 reversed the inhibitory effects of miR-146a on osteogenesis. Together, our results showed that the inflammatory environment mimic TNF*-α* inhibits osteogenesis via upregulation of miR-146a and subsequent downregulation of Smad4, thus suggesting that therapeutic manipulation of miR-146a maybe a potential strategy to improve osteogenesis in the context of osteoporosis.

## Introduction

Osteoporosis, a systemic and multifactorial disease leading to morbidity and mortality among the elderly, is increasing in prevalence worldwide.^1^ Bone fractures due to fragility are the most common consequence of osteoporosis, thus placing the elderly at risk for loss of independence, chronic pain, a need for rehabilitation, and excess mortality, especially when hip fractures are considered.^2^ Moreover, fragility fractures cause significant social, economic, and individual burdens owing to prolonged hospitalizations, medical treatments, limitations in activities of daily life, and demand for healthcare assistance.^3^ Inflammation is elevated in the osteoporosis patients, especially in diabetic and menopausal patients. Accumulating evidence has revealed the negative role of inflammation in osteogenesis of bone mesenchymal stem cells (BMSCs). Among the inflammatory cytokines, TNF*-*α is considered a major factor in osteogenesis inhibition under inflammatory conditions. For example, TNF-α has an extremely broad spectrum of biological activity and has a central role in many inflammatory diseases.^4^ Research has shown that increased TNF-α strongly contributes to the inhibition of osteogenic differentiation, thus resulting in bone loss.^5,6^ Furthermore, TNF-α induces bone destruction by promoting osteoclast differentiation and inhibiting osteoblast differentiation.^7,8^ However, the detailed downstream mechanism of how TNF*-*α leads to osteogenesis defects remains poorly understood.

MicroRNAs (miRNAs) are endogenous small noncoding ~22-nt RNAs that recognize target mRNAs by interacting with recognition sites in the 3ʹ-untranslated regions (UTRs) and subsequently post-transcriptionally repress target gene expression. miRNAs have been reported to have a crucial role in maintaining bone development and metabolism.^9,10^ Recently, several studies have identified the roles of miRNA in regulating BMSC viability, aging, and differentiation. Complex regulation of various miRNAs is critical for the osteogenic differentiation of BMSCs into mature osteoblasts.^11,12^ Multiple miRNAs and their targets have been characterized. For example, miR-153 suppresses the osteogenic differentiation of human BMSCs by targeting bone morphogenetic protein receptor type II.^13^ MiR-21 promotes osteogenic differentiation of BMSCs via the PI3K/β-catenin pathway.^11^ Further identification and characterization of the key miRNAs involved in the regulation of osteogenesis in the context of inflammation would shed light on developing therapeutic strategies for osteoporosis, especially given the targetable traits of miRNAs. Recently, we and others have found that miR-146a is a target of TNF*-*α/NF*κ*B signaling in other cell types, such as myocytes^14^ and astrocytes,^15^ thus suggesting a possible role in regulating osteogenesis of BMSCs under inflammation.

In this study, we tested the effects of TNF*-*α* *on osteogenesis of BMSCs and explored the putative underlying mechanism by focusing on the role of miR-146a. We found that increased miR-146a by TNF-α results in deficient osteogenesis, owing to inhibition of Smad4, a key player in the BMP signal pathway. Our present data suggest that therapeutic manipulation of miR-146a may potentially be a strategy to improve osteogenesis in the context of osteoporosis.

## Materials and methods

### Isolation, culture, and identification of BMSCs

C57Bl6 mice (8-week-old male) were obtained from the Animal Laboratory Center of Sun Yat-sen University, Guangzhou, China. The animals were maintained under specific-pathogen-free conditions and handled in accordance with the NIH Animal Care and Use Committee Regulations. All procedures were in accordance to the Institutional Animal Care and Use Committee. Briefly, total bone marrow was flushed with culture medium from the mouse femora and pelleted before being seeded into 25 cm^2^ culture flasks with complete BMSC culture medium [Dulbecco's modified eagle medium (DMEM)/F12 (1:1; Gibco St. Louis, MO, USA)] supplemented with 10% fetal bovine serum (FBS; Gibco), 1% penicillin/streptomycin(Gibco) at 37 °C with 5% CO_2_. After 3 days of incubation, the non-adherent hematopoietic cells were discarded with the culture medium, and the adherent cells were considered BMSCs. The culture medium was replaced every 3 days thereafter. Approximately 7–9 days after seeding, the cells became nearly 80% confluent. The adherent cells were released from the dishes by using 0.25% trypsin (Gibco) and expanded at a 1:3 dilution. At passages 3–5, BMSCs were used in all subsequent experiments.

For cell characterization, expression of specific cell surface markers for BMSCs were detected by fluorescence activated cell sorting (FACS). CD105, CD90 and CD44 are known markers for BMSCs, and CD45 is a marker for hematopoietic cells. In addition, CD34 is a marker for hematopoietic cells and is partially expressed in certain BMSCs.^15^ Cells were detached by trypsin incubation, rinsed with phosphate buffered saline, and then incubated with fluorescence-labeled antibodies (CD105-FITC, CD90-FITC, CD45-FITC, CD44-FITC or CD34-FITC; BioLegend, San Diego, CA, USA). The antibody-incubated cells were then analyzed using a BD FACS Calibur flow cytometer (BD Biosciences, San Jose, CA, USA).

### Osteogenic, adipogenic, and chondrogenic induction of BMSCs

Osteogenic differentiation was induced by culture with osteogenic medium (complete BMSC culture medium plus 0.1 μmol·L^−1^ dexamethasone, 10 mmol·L^−1^ sodium β-glycerophosphate, and 50 μg·mL^−1^ L-ascorbic acid). The culture medium was replaced every 3 days. After approximately 21 days, cells were stained using an Alizarin Red staining kit (Shanghai, China) for visualization of the calcium nodules.

Adipogenic differentiation was achieved by culture with complete BMSC culture medium supplemented with adipogenic components (1.0 μmol·L^−1^ dexamethasone, 0.5 mmol·L^−1^ 1-methyl-3-isobutyl-xanthine, and 2 mg·L^−1^ insulin). The culture medium was replaced every 3 days. After approximately 14 days, cells were stained with 0.3% Oil Red O for visualization of the lipid droplets. Later, cells were washed with 60% isopropanol to remove excessive nonspecific staining.

Differentiation of BMSCs into chondrocytes was achieved by using complete BMSC culture medium in combination with chondrogenic supplements (50 μg·mL^−1^ L-ascorbic acid, 100 nmol·L^−1^ dexamethasone, 100 μg·mL^−1^ pyruvate, 40 μg·mL^−1^ proline, 50 mg·mL^−1^ ITS +Premix, and 10 ng·mL^−1^ TGF-β1). The culture medium was replaced every 3 days. After approximately 14 days, cells were stained using 0.1% Alcian blue solution.

### MTT assay

The MTT assay for cell proliferation was carried out as described previously.^16^ Cells were grown in 96-well plates in complete BMSC culture medium with and without additional TNF*-α* treatment. The cells were subjected to MTT assays at the indicated time. Cell numbers were calculated on the basis of the optical density values.

### miRNA synthesis and transfection

The control and miR-146a inhibitor, miR-146a mimics, and Smad4 RNAi were synthesized by Genpharm (Shanghai, China). The detailed sequences are shown in Table 1. Transfection was performed using Lipofectamine 2000 (Invitrogen, Carlsbad, CA, USA) according to the instruction manual.

### RNA extraction and qPCR analysis of mRNA/miRNA expression

Total RNA was isolated from BMSCs with TRIzol (Invitrogen). cDNA for mRNA was generated by MMLV (Promega, Madison, WI, USA), and miRNA was produced with a miScript Reverse Transcription Kit (Qiagen, Hilden, Germany), according to the manufacturer’s instructions. Real-time PCR analysis was performed on a Real-Time PCR Detection System (ABI system, Waltham, MA, USA) with a 20 μL reaction containing 2 μL reverse transcription product, 10 μL 2×SYBR Premix Ex Taq^TM^ II (TAKARA, Otsu, Shiga, Japan), 2 μL PCR forward primer (2 μmol·L^−1^, Table 1), 2 μL Universal Adaptor PCR Primer for miRNAs (2 μmol·L^−1^) or 2 μL PCR reverse primer for mRNAs (2 μmol·L^−1^, Table 1), 4 μL ddH_2_O. The PCR conditions involved pre-denaturation at 95 °C for 2 min, followed by 40 cycles, and then ramping up from 66°C to 95 °C to obtain the melting curve. Each sample was analyzed in triplicate. *Gapdh* or *U6b* snRNA was used as a normalization control. Relative expression values from three independent experiments were calculated with the 2^−ΔΔCt^ method.

### Luciferase reporter assay

The putative miR-146a recognition sites in the Smad4 3ʹ-UTR were predicted by TargetScan (Release 6.2, http://www.targetscan.org). The Smad4 3ʹ-UTR reporter plasmid and the mutant form were cloned using the 3'-UTR region of the pGL3-control vector. The reporter vector together with the internal control pRL-TK, control or experimental miRNA mimics/inhibitors was transfected into BMSCs. The relative luciferase activities of the firefly and Renilla luciferase were analyzed with a Dual Luciferase Reporter assay (Promega), per the manufacturer’s instructions. pGL3 BRE Luciferase (Addgene, Plasmid #45126) was obtained from Addgene, which was originally constructed by Martine Roussel *et al.*^17^

### Western blotting

Cells were lysed in RIPA buffer containing Halt™ Protease and Phosphatase Inhibitor Cocktail (Thermo Scientific) at 4 °C. Cell lysates were used for western blot analyses of the protein expression. Briefly, approximately 60 μg total cell lysates were separated by 10% SDS–PAGE gels, then transferred to polyvinylidenedifluoride membrane (Millipore), immunoblotted with the indicated antibodies, and visualized using an enhanced chemiluminescence detection system(Amersham Biosciences).

### Statistical analysis

All data were analyzed using SPSS 19.0 (SPSS Inc., Chicago, IL, USA) and are expressed as the mean±s.d. Comparisons between two groups were performed using Student’s *t*-test, and the significance of differences between three or more experimental groups was determined by one-way analysis of variance. Statistical significance was accepted at *P*<0.05.

## Results

### Isolation and characterization of BMSCs

**Figure 1:**
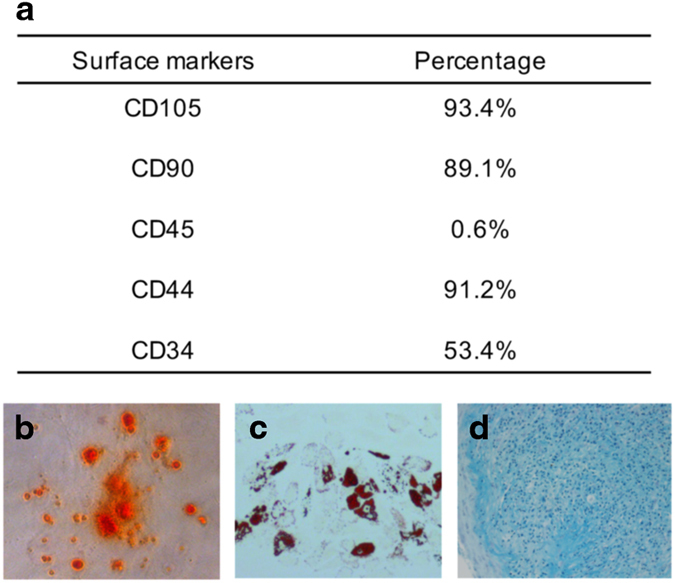
Characterization of BMSCs. (**a**) The expression of cell surface markers for BMSCs, as detected by flow cytometry. The differentiation capability of BMSCs into osteogenic, adipogenic, or chondrogenic cells was evaluated by Alizarin Red S staining (**b**), Oil Red O staining (**c**) and Alcian blue staining (**d**).

To confirm the identity of the isolated BMSCs, the cells were first characterized by flow cytometry to detect surface marker expression. As shown in Figure 1a, the cultured BMSCs were approximately 90% positive for mesenchymal markers, such as CD105, CD90, and CD44, and negative for hematopoietic cell surface markers, such as CD45. Notably, approximately half of the isolated BMSCs also expressed CD34 (Figure 1a), thus further suggesting the heterogeneous characteristics of the BMSC population. Moreover, these MSCs were able to be differentiated into osteoblasts, adipocytes, and chondrocytes under standard *in vitro* differentiating conditions, as determined by Alizarin Red S staining, Oil Red O staining, and Alcian blue staining, respectively (Figure 1b–d). These data confirmed the identity of the isolated and cultured BMSCs.

### Effects of TNF*-*α on the proliferation and osteogenic differentiation of BMSCs

**Figure 2:**
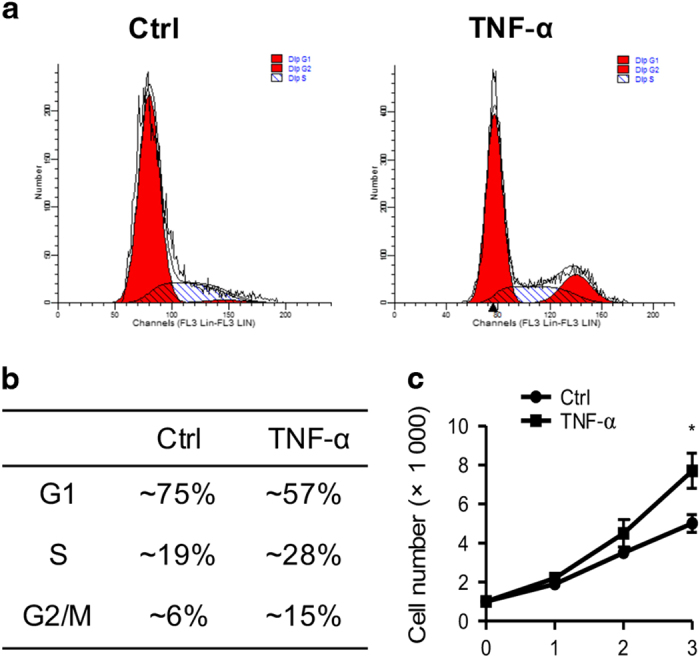
TNF-α promotes BMSC proliferation. (**a**) BMSCs were cultured with or without TNF*-*α* *(10 ng·mL^−1^), and cell cycle distribution was analyzed by flow cytometry. (**b**) Quantification data of (**a**). (**c**) Cell survival and proliferation were analyzed by MTT assays. Experiments were performed at least in triplicate. *P<0.05.

Given that inflammation is one of the most important causes of osteoporosis, we tested whether TNF*-*α treatment could mimic the inflammatory microenvironments. There were more cells in S phase after TNF*-*α treatment, as compared with the control, as revealed by flow cytometry (Figure 2a and b). Accordingly, MTT assays further confirmed that TNF*-*α promoted cell proliferation (Figure 2c).

**Figure 3:**
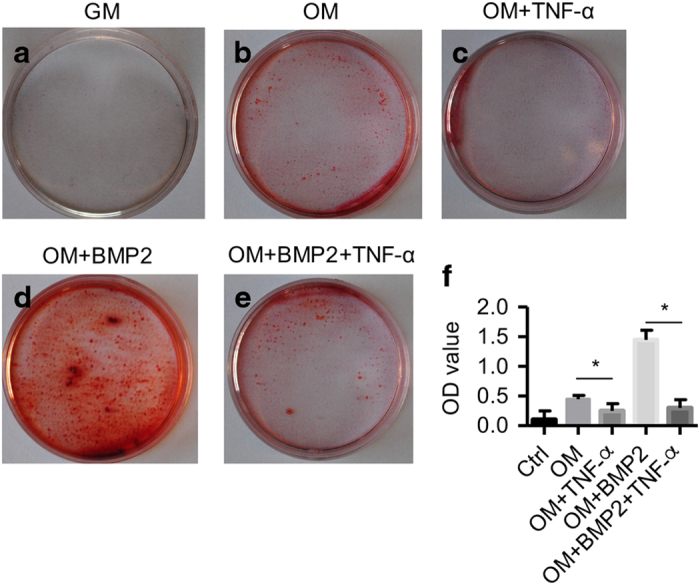
Inflammation inhibits osteogenesis of BMSCs. BMSCs were cultured in different conditions, then stained with Alizarin Red S. Control conditions: (**a**) osteogenic differentiation medium without BMP2; (**b**) osteogenic differentiation medium without BMP2 and with TNF-α (10 ng·mL^−1^); (**c**) osteogenic differentiation medium combined with BMP2 (50 ng·mL^−1^); (**d**) osteogenesis medium combined with BMP2 (50 ng·mL^−1^); and inflammatory stimulation with TNF*-*α (10 ng·mL^−1^) (**e**). Quantification of the Alizarin Red staining results in the above groups (**f**). GM, growth medium; OM, osteogenic medium with 50 ng·mL^−1^ BMP2.

In contrast to the proliferation-promoting role of TNF*-*α, Alizarin Red staining indicated that TNF*-*α (10 ng·mL^−1^) inhibited osteogenic differentiation (Figure 3a–e). Notably, the inhibitory effects were much stronger when BMP2 (50 ng·mL^−1^) was added as a stimulator, as observed in the relative fold change of the Alizarin Red staining (Figure 3e).

### TNF*-*α inhibits osteogenesis in a miR-146a-dependent manner

**Figure 4:**
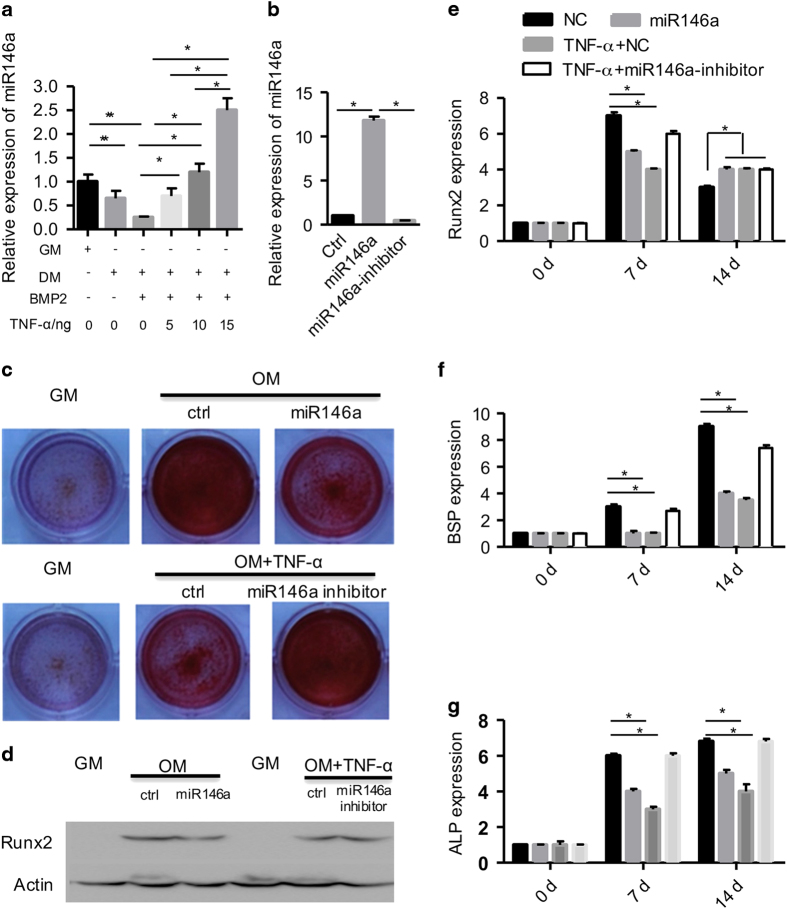
TNF*-*α inhibits osteogenesis in a miR-146a-dependent manner. (**a**) Osteogenic differentiation decreasesmiR-146a expression, and TNF-α dose dependently increases miR-146a expression. (**b**) Overexpression and knockdown efficiency of miR-146a. (**c**) BMSCs were treated as indicated, and osteogenesis was observed by Alizarin Red staining. GM, growth medium; OM, osteogenic medium with BMP2 (50 ng·mL^−1^). (**d**) BMSCs were treated as indicated, and osteogenesis was observed by western blot analysis of Runx2. (**e**–**g**) The expression of Runx2 (**e**), BSP (**f**) and ALP (**g**) at the RNA level was analyzed by qPCR. *n*≥3, *P<0.05.

To further explore the underlying mechanism of how TNF-α inhibits osteogenesis, we focused on the possible involvement of miR-146a. Quantitative real-time PCR analysis revealed that endogenous expression of miR-146a expression decreased after osteogenic differentiation, but was increased by TNF*-*α in a dose-dependent manner(Figure 4a). We next transfected the differentiating BMSCs with a control or miR-146a mimic or inhibitor. Quantitative PCR analysis confirmed the forced expression and knockdown efficiency of miR-146a mimics and inhibitors (Figure 4b). Moreover, overexpression of miR-146a decreased the calcium nodules, as shown by Alizarin Red staining, whereas inhibition of miR-146a rescued the TNF*-*α-mediated repression of osteogenesis (Figure 4c). Accordingly, quantitive PCR analysis revealed that miR-146a inhibited Runx2, BSP and ALP expression (Figure 4e–g), whereas knockdown of miR-146a restored their expression under TNF*-*αtreatment, thus further confirming the findings from the Alizarin Red S staining.

### Increased miR-146a inhibits Smad4 expression through the 3ʹ-UTR

**Figure 5:**
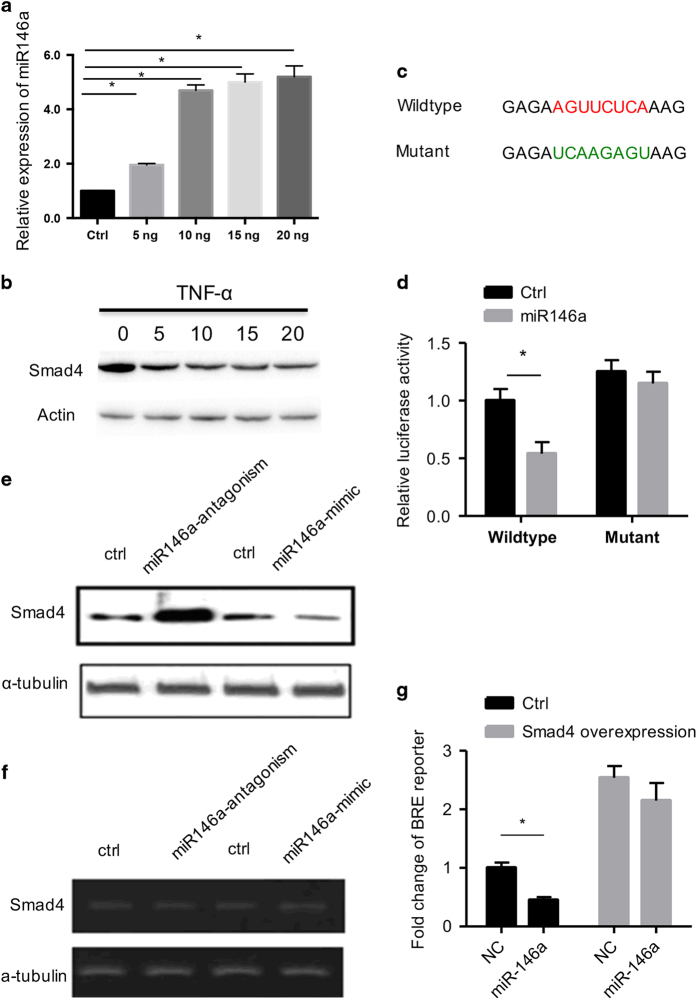
Increased miR-146a inhibits Smad4 expression through the 3ʹ-UTR. (**a**) The expression of miR-146a was examined by RT-PCR in osteogenic medium with different doses of TNF-α (0, 5, 10, 15, and 20 ng). (**b**) The expression of Smad4 was examined by western blot analysis in cells treated as described above. (**c**) Wild-type Smad4 3ʹ-UTR bears a potential miR-146a binding site, which was mutated in the mutant reporter. (**d**) Effects of miR-146a expression on the reporter luciferase activity. Fold change of luciferase activity was calculated and expressed as the mean ±s.d. (*n*=3). *P<0.05. (**e**) Expression of Smad4 at the protein level was analyzed by western blotting after miR-146a overexpression or inhibition. (**f**) Expression of Smad4 at the mRNA level was analyzed by RT-RCR in cells treated as described above. (**g**) BMP reporter luciferase assay. BMP2-stimulated cells were additionally treated as indicated, and the relative luciferase activity of the BMP reporter pGL3 BRE was analyzed. *P<0.05, *n*=3.

The above studies revealed an essential role of miR-146a in the inhibitory effects of TNF*-*α on osteogenesis. Bioinformatics analysis with TargetScan revealed that Smad4, an essential regulator of the BMP signal pathway, might be a target candidate of miR-146a. In agreement with the dose-dependent increase of miR-146a by TNF*-*α, Smad4 expression gradually decreased with increased TNF*-*α concentration (Figure 5a and b). In addition, the endogenous Smad4 expression was decreased by miR-146a overexpression and enhanced by the miR-146 inhibitor (Figure 5e). Strikingly, there were no obvious changes in Smad4 mRNA after overexpression or knockdown of miR-146a (Figure 5f).To clarify the detailed mechanism how miR-146a regulates Smad4, the 3ʹ-UTR reporter and its mutant form were constructed (Figure 5c). Luciferase reporter data revealed that miR-146a inhibited the wild-type reporter activity but had no effect on the mutant form (Figure 5d). To further confirm the role of miR-146a in BMP signaling, the BMP signal luciferase reporter pGL3 BRE Luciferase was assessed. Our results indicated that miR-146a inhibited BMP2 reporter luciferase activity, whereas restoration of Smad4 significantly rescued the inhibition (Figure 5g). Overall, these data indicated that miR-146a inhibits BMP signaling by repressing Smad4 expression through interaction with the 3ʹ-UTR in a post-transcriptional regulatory mechanism.

### Restoration of Smad4 rescues the effects of miR-146a on osteogenesis

**Figure 6:**
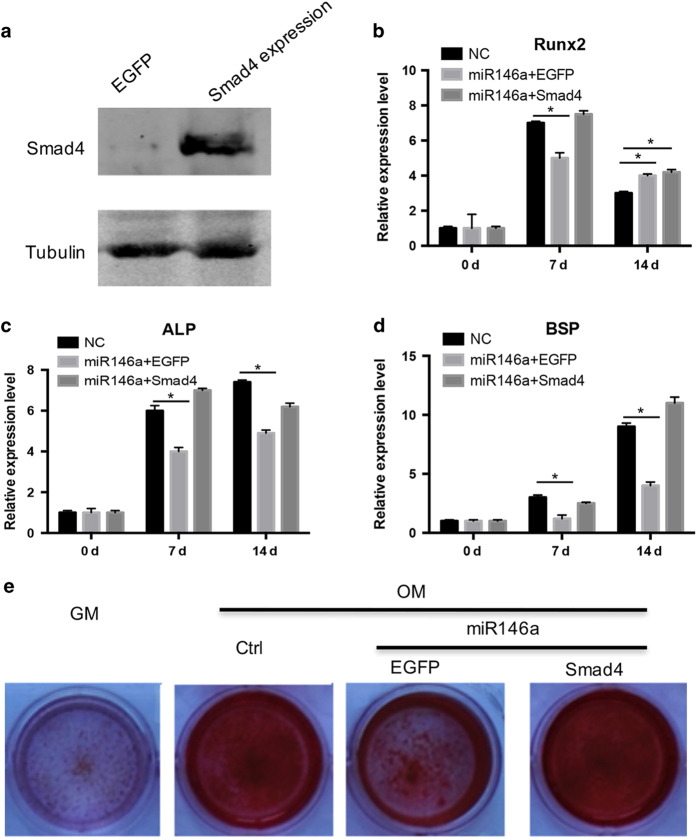
Restoration of Smad4 blocks the inhibitory effects of miR-146a on osteogenesis. (**a**) BMSCs were infected with control or lentivirus expressing Smad4, and the expression of Smad4 at the protein level was analyzed by western blotting. (**b**–**d**) BMSCs were transfected with miR-146a together with control EGFP or Smad4, and this was followed by osteogenic medium culture (osteogenic medium plus 50 ng·mL^−1^ BMP2). The expression of Runx2 (**b**), ALP (**c**), and BSP (**d**) at the mRNA level was analyzed by qPCR. *n*≥3, *P<0.05. (**e**) Cells were treated as described above, and calcium salt nodules were detected by Alizarin Red staining. GM, growth medium; OM, osteogenic medium with 50 ng·mL^−1^ BMP2. Data presented are representative of three experiments.

The above analysis established the existence of a TNF-α-miR-146a-Smad4 regulatory axis in the BMSCs under inflammatory conditions. To further confirm whether downregulation of Smad4 contributes to the TNF*-*α-miR-146a axis-related osteogenesis defects, we restored Smad4 expression in the context of miR-146a overexpression. Smad4 overexpression was conducted by infection of a Smad4-overexpressing lentivirus (Figure 6a). After Smad4 restoration, the inhibitory effects of miR-146a on the expression of Runx2, ALP and BSP were also rescued (Figure 6b–d). In accordance with the osteogenic gene expression, Alizarin Red staining results also confirmed the effects of miR-146a-Smad4 on osteogenesis (Figure 6e).

## Discussion

Identification of the key players responsible for osteoporosis would contribute to prevention and therapeutic efforts. In this study, we confirmed the inhibitory effects of TNF*-*α on osteogenesis of BMSCs and demonstrated that the TNF*-*α-miR-146a -Smad4 axis contributes to osteogenesis defects. Our data suggested that therapeutic manipulation of miR-146a maybe a potential strategy to improve osteogenesis in the context of osteoporosis.

Multiple miRNAs, such as miR26a, miR153, and others,^13,18,19^ have been found to have important roles in osteogenesis by fine-tuning the osteogenesis-related genes. The miRNAs themselves are also believed to be regulated by multiple growth factors, cytokines and hormones. miR-146a is a well-known target of inflammation. Our study provides an example of the link between cytokines/hormones/growth factors and osteogenesis. miR-146a may not be the only miRNA involved in TNF*-*α-related osteogenesis defects, and thus, other miRNAs might be involved.

In our study, we found that miR-146a inhibits osteogenesis defects by targeting Smad4. Smad4 may not be the only target of miR-146a. Other downstream targets might be also involved in the function of miR-146a, given that miRNAs usually have multiple targets. As suggested by previous results, miR-146a has been identified as a negative feedback regulator of NF-*κ*B activation,^20,21^ which is also considered to be an inhibitor of osteogenic differentiation of BMSCs. It has also been reported that miR-146a regulates osteogenic differentiation by downregulating SMAD2 and SMAD3 in human fetal femur-derived skeletal stem cells.^22^ All these data clearly support the osteogenesis inhibitory role of miR-146a. miRNAs function as potent molecular managers that may simultaneously regulate multiple endogenous signaling pathways, and miRNAs and RNAi have been proposed as rational gene-specific therapies for certain disease. We here revealed that miR-146a functions as a potent osteogenesis repressor by decreasing Smad4 expression. Thus, inhibition of miR-146a may promote bone regeneration *in vivo*. Notably, the BMSCs used in this study were bulk cells rather than sorted stem cells, and thus, future experiments with sorted BMSCs would further strengthen this conclusion, although most of the unsorted cells were BMSC marker positive, as shown in Figure 1. Notably, previous *in vitro* studies have suggested that the inhibitory effect of TNF*-*α on osteoblast differentiation results from inhibition of IGF-1 (ref. 23) and the key osteoblast differentiation transcription factors Osterix^24^ and Runx2.^25^ Thus, it is of interest to determine whether the TNF*-*α-miR-146a-Smad4 axis directly alters the expression of IGF-1, Osterix and/or Runx2 at the transcriptional and/or post-transcriptional levels. Specifically, it would be of interest to search for Smad4 response elements in the promoter regions and miR-146a recognition sites in the 3ʹ-UTR region of these genes.

It is now widely recognized that developing effective delivery systems is key to miRNA/RNAi-based therapy.^26^ Primary stem cells are more difficult to transfect than immortalized cell lines, and thus, delivery of miRNAs in the context of bone regeneration maybe a major obstacle for future experimental and clinical studies. In addition to low cytotoxicity and high transfection efficiency, an ideal miRNA delivery system for regenerative medicine should be able to deliver the miRNAs to specific cells, tissues or organs in a local and sustained manner.^27^ Targeted delivery of miR-146a or other potential miRNAs *in vivo*, which may provide a way to maximally mimic the native bone development environment, is underway and would open an avenue for osteoporosis and related disease therapy. Thus, further clarification of the status of the miR-146a-Smad4 axis in clinical osteoporosis patients or in an ovariectomized mouse model would shed light on targeting this axis as a potential therapeutic strategy.

## Conclusion

In this study, we provide the first evidence that^1^ miR-146 expression of BMSCs is significantly increased under TNF-*α* treatment. Increased miR-146a expression in turn inhibits osteogenesis, thus at least partially explaining how inflammation augments osteoporosis.^2^ miR-146a decreases osteogenic differentiation by post-transcriptionally downregulating Smad4, an essential mediator of the BMP pathway. Further clinical analysis of miR-146a-Smad4 in patients with osteoporosis should demonstrate the clinical relevance of the current study and thus shed light on osteoporosis prevention and therapy.

## Figures and Tables

**Table 1:** Sequences of the primers or miRNA inhibitors/mimics used in the study

Names	Forward primers (5ʹ-3ʹ)	Reverse primers (5ʹ-3ʹ)
Runx2	ACACCGTGTCAGCAAAGC	GCTCACGTCGCTCATCTTG
Alp	AACAACCTGACTGACCCTTCG	AATCCTGCCTCCTTCCACC
Bsp	GTCTTTAAGTACCGGCCACG	TGAAGAGTCACTGCCTCCCT
Smad4	TCACTATGAGCGGGTTGTCTC	TCCTTCAGTGGGTAAGGACG
*Gapdh*	CGTCCCGTAGACAAAATGGT	TTGATGGCAACAATCTCCAC
*miR-146a*	TGAGAACTGAATTCCATGGG	Universal primer provided
*U6*	CTCGCTTCGGCAGCACA	AACGCTTCACGAATTTGCGT
*NC mimics*	UUCUCCGAACGUGUCACGUTT	ACGUGACACGUUCGGAGAATT
*miR-146a mimics*	UGAGAACUGAAUUCCAUGGGUU	CCCAUGGAAUUCAGUUCUCAUU
*NC inhibitor*	UUCUCCGAACGUGUCACGUTT	
*miR-146a inhibitor*	AACCCAUGGAAUUCAGUUCUCA	
